# Relationships of oxidative stress, inflammation and gut microbiota with cognitive impairment in first-episode major depressive disorders: a pilot study in China

**DOI:** 10.3389/fmicb.2026.1842145

**Published:** 2026-07-07

**Authors:** Hehua Li, Baoyuan Zhu, Yuanyuan Huang, Shuhao Chen, Ziyun Zhang, Jingping Wu, Fengchun Wu, Kai Wu, Yuping Ning

**Affiliations:** 1 The First School of Clinical Medicine, Southern Medical University, Guangzhou, China; 2 The Affiliated Brain Hospital, Guangzhou Medical University, Guangzhou, China; 3 School of Materials Science and Engineering, South China University of Technology, Guangzhou, China; 4 School of Biomedical Sciences and Engineering, South China University of Technology, Guangzhou International Campus, Guangzhou, China; 5 Guangdong Engineering Technology Research Center for Translational Medicine of Mental Disorders, Guangzhou, China; 6 Key Laboratory of Neurogenetics and Channelopathies of Guangdong Province and the Ministry of Education of China, Guangzhou Medical University, Guangzhou, China

**Keywords:** cognitive function, gut microbiota, major depressive disorder, metabolic pathway, peripheral blood indicators

## Abstract

**Objective:**

Cognitive impairment runs through the entire course of major depressive disorder (MDD). However, the relationships between cognitive impairment and the gut microbiota (GM) and their predicted metabolic pathways as well as peripheral blood indicators remains unclear. We aimed to explore these relationships.

**Method:**

Patients (*n* = 61) and healthy controls (HCs, *n* = 84) were enrolled. Our analyses were performed using data from the Hamilton Depression Scale, cognitive function (MATRICS™ Consensus Cognitive Battery [MCCB]), the GM and their predicted metabolic pathways, and peripheral blood indicators, including homocysteine (Hcy), superoxide dismutase (SOD), and C-reactive protein (CRP).

**Results:**

In comparison with HCs, patients with MDD exhibited significant cognitive impairment, elevated SOD levels, enrichment of specific GM, and upregulation of microbial predicted metabolic pathways involving L-alanine, pyruvate, and salicortin. In patients with MDD, the salicortin biosynthesis pathway and pathways related to L-alanine metabolism were negatively correlated with the levels of Hcy and CRP, respectively, while the superpathway of *de novo* pyrimidine deoxyribonucleotide biosynthesis was positively correlated with the SOD levels. The abundance of *Blautia_caecimuris* and *Dysosmobacter_*sp.*_NSJ-60* was positively correlated with the scores for processing speed and attention/vigilance domain, while the abundance of *Enterocloster_aldenensis* was negatively correlated with the score for working memory. Moreover, the 6-gingerol analog biosynthesis pathway was negatively correlated with the score for processing speed.

**Conclusion:**

Our research showed that the GM and their predicted metabolic pathways in patients with MDD were closely related to cognitive function and peripheral blood indicators, and that differences in these factors may manifest as oxidative stress and inflammation.

## 1 Introduction

Cognitive impairment, which is defined as “diminished ability to think or concentrate, or indecisiveness” according to the Diagnostic and Statistical Manual of Mental Disorders (DSM-5), is a fundamental characteristic of major depressive disorder (MDD) (Regier et al., 2013). Approximately 26% of individuals with first-episode MDD exhibit cognitive dysfunction (Vicent-Gil et al., 2018), and patients experiencing recurrent episodes demonstrate substantially higher rates of cognitive impairment (Conradi et al., 2011). Preservation of cognitive function is a critical determinant for restoring psychosocial functioning in patients with MDD. Cognitive deficits frequently persist beyond the remission of affective symptoms (Bortolato et al., 2016). Furthermore, clinical evidence suggests that depressed patients with cognitive impairment show significantly elevated risks for suicidal behavior and self-harm (Martinez-Aran et al., 2009). However, the existing first-line antidepressant medications achieve symptomatic remission in only 30%–50% of patients (Zhdanava et al., 2021). Moreover, drugs that can address the cognitive and social functions of patients through different mechanisms are currently lacking. Therefore, research on the cognitive function of patients with MDD requires more attention.

The gut microbiota is associated with the central nervous system through multiple pathways, and these interactions are mediated by a “microbiota-gut-brain axis” (Wu et al., 2026; Zhu et al., 2026). The gut microbiota play key roles in the occurrence and development of various neuropsychiatric diseases, including Parkinson disease (Grant et al., 2023), Alzheimer’s disease (Chandra et al., 2023), MDD (Liu et al., 2025), autism (Petra et al., 2015). Previous studies using metagenomic approaches have demonstrated disruptions in the gut microbiota in patients with MDD (Yang et al., 2020). Furthermore, fecal microbiota transplantation from healthy adolescent donor mice into adolescent mice showing depression induced by chronic restraint stress has been shown to significantly ameliorate depressive-like behaviors (Zhou et al., 2023). These findings highlight the important role of the gut microbiota in the pathogenesis and progression of MDD. Nevertheless, research exploring the association between gut microbiota alterations and cognitive impairments in MDD remains limited. Previous investigations utilizing 16S rRNA gene sequencing to analyze the gut microbiota of patients with MDD revealed a close correlation between microbial changes and cognitive function. Nevertheless, the relatively limited resolution of this method precluded detailed characterization of the specific bacterial species and their predicted metabolic pathways associated with MDD. A recent study attempted to address this limitation by employing metagenomic sequencing alongside 16S rRNA gene sequencing (Dong et al., 2023; Liu P. et al., 2021). However, the MDD patient cohort in that study included individuals who had been receiving various antidepressant medications, which may have confounded microbial composition (Dong et al., 2023). Moreover, the latest research has found that different abundances of *Amycolatopsis* sp. Hca4 in patients with first-episode MDD can regulate the relationship between working memory and functional connectivity of the middle frontal gyrus and parahippocampal gyrus (Huang et al., 2025), the mechanism by which the gut microbiota affects the brain remains unclear. Therefore, more research is required to strengthen the understanding of the relationship between gut microbiota alterations and cognitive impairment in patients with MDD.

Blood is a carrier for metabolic products and acts as a bridge facilitating crosstalk between the brain and the gut microbiota (Guest et al., 2016). Inflammation in the peripheral blood can induce changes in the human brain, thereby influencing mood or cognitive function (Guest et al., 2016). And reactive oxygen species (ROS) in the peripheral blood play a crucial role in cellular signaling and defense against invading microorganisms. Excessive ROS production and depletion of antioxidant defenses can trigger pro-inflammatory signaling, damage essential macromolecules, and induce apoptosis. The inability of cells to maintain redox homeostasis, along with the subsequent generation of pro-inflammatory mediators, can lead to cellular necrosis. Due to its high oxygen consumption, high lipid content, and relatively weak antioxidant defenses, the brain is particularly vulnerable to oxidative stress (OS) (Bhatt et al., 2020). Homocysteine (Hcy) is a nonprotein amino acid which may attach a sulfide group to proteins (Jakubowski, 2019). And sulfhydryl radicals bind readily to proteins and cause oxidative damage (Rajendiran et al., 2015). Under OS, Hcy is easily oxidized through the generation of reactive oxygen species (Loscalzo, 1996). Excessive levels of Hcy may have neurotoxic effects and exacerbate OS (Gu et al., 2012). OS is a major contributor to neurodegeneration, and it has been shown to be involved in the pathogenesis of MDD (Harsanyi et al., 2022). Furthermore, the oxidative stress mediated by the gut microbiota can lead to dysfunction of neural rocesses (Shandilya et al., 2022). The metabolites of the gut microbiota, especially short-chain fatty acids, affect mitochondrial function and reduce the production of ROS (Mottawea et al., 2016). In preclinical studies, it was found that the complex interaction between the gut microbiota and OS is related to cognitive function (Mao et al., 2021). Studies on schizophrenia have shown that the OS indicator superoxide dismutase (SOD) is closely related to the gut microbiota, and the gut microbiota is positively correlated with cognitive functions such as visual learning and processing speed. However, the mechanisms by which alterations in the gut microbiota in MDD interact with OS and inflammation in the peripheral blood to contribute to the cognitive impairment in MDD require elucidation.

Therefore, this study aimed to investigate, for the first time, the relationships of the gut microbiota, their predicted metabolic pathways, and peripheral blood markers with the clinical symptoms and cognitive function in patients with MDD. Using fecal samples as well as the data for cognitive indicators and peripheral blood markers of inflammation and OS in first-episode drug-naïve patients with MDD and matched healthy controls (HCs), our study aimed to elucidate that the levels of peripheral blood inflammatory and oxidative stress markers can act on cognitive impairment through the abnormal effects of specific gut microbiota and their predicted metabolic pathways. The findings are expected to enhance the understanding of the pathophysiology of cognitive deficits in MDD, guide clinical management of patients with existing cognitive impairments, and provide a theoretical basis for exploring novel therapeutic and preventive strategies for MDD-related cognitive dysfunction.

## 2 Methods

### 2.1 Experimental design and participant recruitment

In this cross-sectional case–control study, all patients with MDD were from the psychiatric outpatient or inpatient department of the Affiliated Brain Hospital of Guangzhou Medical University, and HCs were recruited from the community through posters and push by WeChat Moments. This study was approved by the Ethics Committee of the Affiliated Brain Hospital, Guangzhou Medical University and was conducted in strict accordance with the Declaration of Helsinki. All participants provided informed consent before participating in the experiment. The inclusion criteria for the patient group were as follows: ① meeting the DSM-5 diagnostic criteria for MDD; ② age from 18 to 45 years, with more than 6 years of education; ③ presenting with the first attack (disease course ≤2 years) and no use of psychiatric drugs; ④ and Hamilton Depression Scale-17 (HAMD-17) score ≥17 points. The inclusion criteria for the HC group were as follows: ① aged 18–45 years, with more than 6 years of education; ② no history of mental disorders meeting the DSM-5 criteria for any mental disorders; and ③ no family history of mental disorders. The exclusion criteria for both groups were as follows: ① presence of other severe mental disorders meeting the corresponding DSM-5 diagnostic criteria, including schizophrenia, bipolar disorder, organic mental disorder, substance abuse disorder, and intellectual disability; ② severe physical diseases, including infectious diseases, immune system diseases, and nervous system diseases; ③ history of head trauma or disturbance of consciousness; ④ severe diarrhea or use of antibiotics in the past 3 months; or ⑤ pregnant or lactating women.

The basic information and clinical indices, including sex, age, years of education, and body mass index (BMI), of all participants are recorded in Table 1. To control confounding factors, since obesity is closely related to inflammation and OS, the two groups also underwent matching for BMI in addition to matching for sex, age, and years of education. And the patients also need to be collect duration of disease and clinical symptoms. Fecal and blood samples of all participants were collected in the morning after at least 8 h of fasting; the fecal samples were stored at −80 °C for further processing, while the blood samples were sent to the laboratory medicine department for biochemical evaluations.

**Table 1:** Comparison of clinical characteristics between the two groups.

Variable	MDD	HC	X2/T/Z	*p*
Sex (male/female)	29/32	36/48	*0.313*	0.576
Education (years)	15.0 ± 1.8	15.4 ± 1.7	−1.551	0.123
Age (years)	22.3 ± 3.1	22 ± 2.5	0.664	0.508
BMI (kg/m^2^)	21.1 ± 3.8	21.5 ± 3.8	−0.706	0.482
Disease duration (month)	12.5 ± 9.8	—	—	—
CGI	5.3 ± 0.8	—	—	—
HAMD	22.3 ± 4.0	—	—	—
Processing speed	34.1 ± 10.0	45.6 ± 10.1	−6.864	<0.001
Attention/vigilance	35.4 ± 10.1	42.7 ± 8.5	−4.752	<0.001
Working memory	41.6 ± 11.7	46.6 ± 11.2	−2.602	0.010
Verbal learning	37.1 ± 9.3	41.6 ± 8.1	−3.120	0.002
Visual learning	42.7 ± 7.3	45.4 ± 7.1	−2.149	0.033
Homocysteine	14.4 ± 6.4	14.4 ± 10.2	0.002	0.998
C-reactive protein	1.3 ± 2.6	1.7 ± 3.7	−0.628	0.531
Superoxide dismutase	190.9 ± 16.7	184.6 ± 17	2.201	0.029

BMI: body mass index, CGI: clinical global impression scale, HAMD: Hamilton depression scale score.

### 2.2 Clinical data assessment

HAMD-17 was used to assess the severity of depressive symptoms in the patient group, and a total score ≥17 was considered to indicate depressive symptoms. Drawing on prior recommendations from cognitive assessment studies in patients with affective disorders (Ahern and Semkovska, 2017), the MATRICS™ Consensus Cognitive Battery (MCCB) was used to comprehensively assess the cognitive function of all subjects. The MCCB includes seven cognitive dimension assessments, of which we employed five for the reason that Meta-analyses have consistently shown that the first-episode drug-naïve patients with MDD exhibit these five aspects of cognitive impairments (Lee et al., 2012), namely, processing speed, attention/vigilance, working memory, verbal learning, and visual learning (Huang et al., 2025). The MCCB software used the Chinese norm to adjust the output for age, sex, and education level, and used the standard T score for statistical analysis. Higher scores indicated better cognitive function. Two trained and qualified professionals performed clinical assessments for all participants, and the internal consistency was >80%.

### 2.3 Detection of peripheral blood indicators

A 5-mL peripheral blood sample was collected from each participant after at least 8 h of fasting. Blood collection was scheduled between 7:00 and 9:00 a.m. After collection, the samples were transported to the Central Laboratory of the Affiliated Brain Hospital of Guangzhou Medical University for serum/plasma separation. Subsequent analyses were performed promptly after processing. The measured parameters included the homocysteine (Hcy) level, superoxide dismutase (SOD) activity, and C-reactive protein (CRP) level. Hcy levels were measured using an enzymatic assay with the Homocysteine Assay Kit (Beijing Strong Biotechnologies Co., Ltd., Beijing, China). SOD activity was determined by the pyrogallol autoxidation method using a kit provided by Fujian Fuyuan Biotechnology Co., Ltd., Fujian Fuyuan, China. CRP levels were quantified by immunoturbidimetry employing the CRP detection kit (Beijing Leadman Biochemistry Co., Ltd., Beijing, China.).

### 2.4 Collection of fecal samples and extraction, processing, and analysis of DNA

Fecal samples (approximately 200 mg) were collected from the mid- and posterior segments of fresh stool, placed in sterile, airtight containers, and immediately frozen at −80 °C for subsequent DNA extraction, processing, and analysis. The detailed protocol is provided in the following paragraphs.

The fecal samples were first subjected to metagenomic sequencing and quality control. The Agencourt SPRIselect (Beckman Coulter, USA, Catalog #: 2358413) was used to extract DNA from the fecal samples. The fresh genomic DNA samples were mechanically fragmented to ~400 bp with Bioruptor Pico (Diagenode, Belgium). A magnetic bead-based method was used for selection of DNA fragments in accordance with a standard protocol (Agencourt AMPure XP). Libraries were prepared by using the NEBnext Ultra II DNA Library Prep Kit for Illumina (New England BioLabs). The Illumina HiSeq X Ten platform was then used for 2 × 150 bp paired-end whole-metagenome sequencing. To ensure the accuracy and reliability of the subsequent analysis, FastQC software was used to evaluate the quality of sequencing data by multi-dimensional analysis of read quality, base composition, GC content, and repeatability. Fastp was used to control the quality of the offline data, trim low-quality reads and splice sequences, and correct erroneous sequences. The KneadData toolkit, which integrates Trimmomatic software for sequence quality control and Bowtie2 software to align sequencing data with host gene sequences, was used for sequence quality control and host sequence removal. This toolkit identified and filtered host sequences and potentially contaminated sequences to obtain high-quality microbial metagenome sequencing results.

Subsequently, analyses of the microbial community composition as well as functional annotation were performed using the metagenomic data (Peng et al., 2025). MetaPhlAn4 was used to analyze the species-level microbial community composition on the basis of high-quality sequencing data. This tool uses Bowtie2 to efficiently align sequencing data to its database by aligning species-specific marker genes and calculating the coverage of each marker gene. On the basis of these coverage data, MetaPhlAn4 estimated the abundance of each microbial group in the sample, and obtained the relative abundance of species by normalization. Finally, MetaPhlAn4 output the species composition table, which details the relative abundance information of the different microbial species in the sample.

### 2.5 Statistical analysis

Statistical analyses of demographic data as well as information from clinical assessments and peripheral blood measurements were performed using IBM SPSS Statistics (Version 22.0). Categorical variables were compared between the MDD and HC groups by using Chi-square tests, while continuous variables were analyzed with independent-sample t-tests. All microbiome-related analyses were conducted in R version 4.3.2 using multiple R packages. Specifically, microbial composition analyses and diversity analyses were mainly performed using the phyloseq, vegan, microbiome, and ggplot2 packages. Before alpha-diversity and downstream statistical analyses, low-prevalence taxa were filtered following a standard microbiome preprocessing workflow. Only taxa detected in at least 20% of samples were retained to reduce sparsity caused by rare taxa. All other analysis parameters were set to default values. We used the “vegan” package to perform Alpha diversity analysis, calculating the Shannon and Simpson indices; for Beta diversity analysis, we used the “betadisper” and “adonis” functions from the “vegan” package to assess microbiome community differences between the MDD and healthy control groups based on Bray-Curtis distances. To evaluate correlations between microbiome features, clinical indicators (such as Hcy, SOD, CRP) and cognitive function, we used the “rcorr” function from the “Hmisc” package to calculate Spearman correlation coefficients, and visualized these correlations using the “corrplot” package. Additionally, for differential analysis, we employed “LEfSe” (Linear Discriminant Analysis Effect Size) to identify significantly different microbial species between the MDD and control groups. And the bar chart is mainly used to present relative abundance variation patterns of selected taxa. For metabolic pathway analysis, we used the `Humann3` tool to generate metabolic pathway data. This tool allows the annotation and quantification of microbial predicted metabolic pathways based on genomic data, providing us with data on the relationship between microbiome features, clinical biomarkers (such as Hcy, CRP, and SOD), and cognitive function. A two-sided significance level of *α* = 0.05 was applied for all tests.

## 3 Results

### 3.1 Clinical characteristics of participants

The two groups showed no differences in sex, years of education, age, BMI, and the serum levels of Hcy and CRP (all *p* > 0.05; Table 1). The scores of all dimensions of the MCCB in patients with MDD were lower than those in HCs (all *p* < 0.05; Table 1). In addition, the serum levels of SOD in patients were much higher than those in HCs (*p* = 0.029; Table 1).

### 3.2 Microbial structure in the two groups

**Figure 1:**
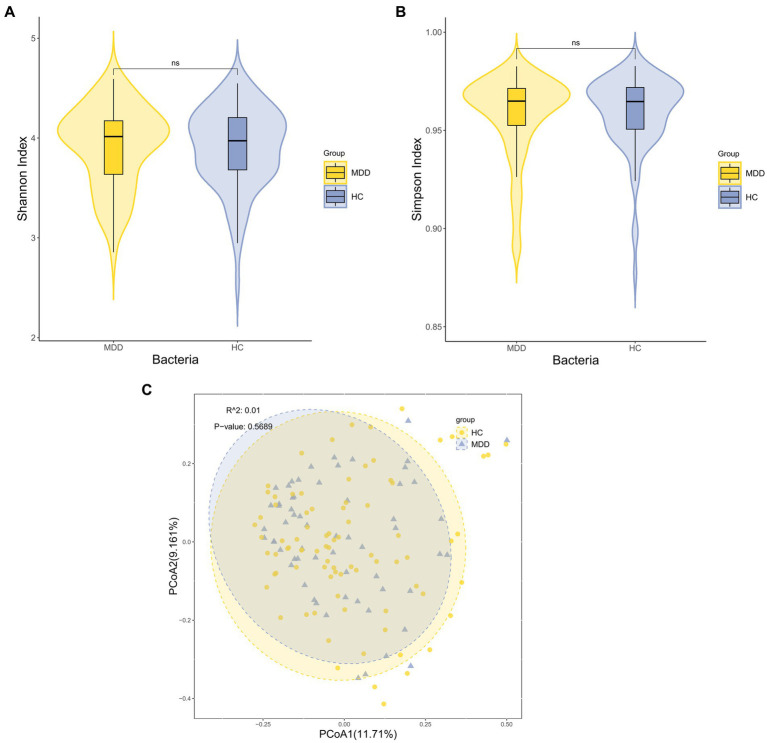
Comparison of the gut microbiome structures. **(A)** Shannon index between MDD and HC group; **(B)** Simpson index between MDD and HC group; **(C)** Beta diversity between MDD and HC group. NS: no significance.

The MDD and HC groups showed no significant difference in the alpha diversity based on the Shannon index and Simpson index and the intergroup *β* diversity based on the Bray-Curtis distance (all *p* > 0.05; Figure 1).

### 3.3 Differences in gut microecology between the two groups

**Figure 2:**
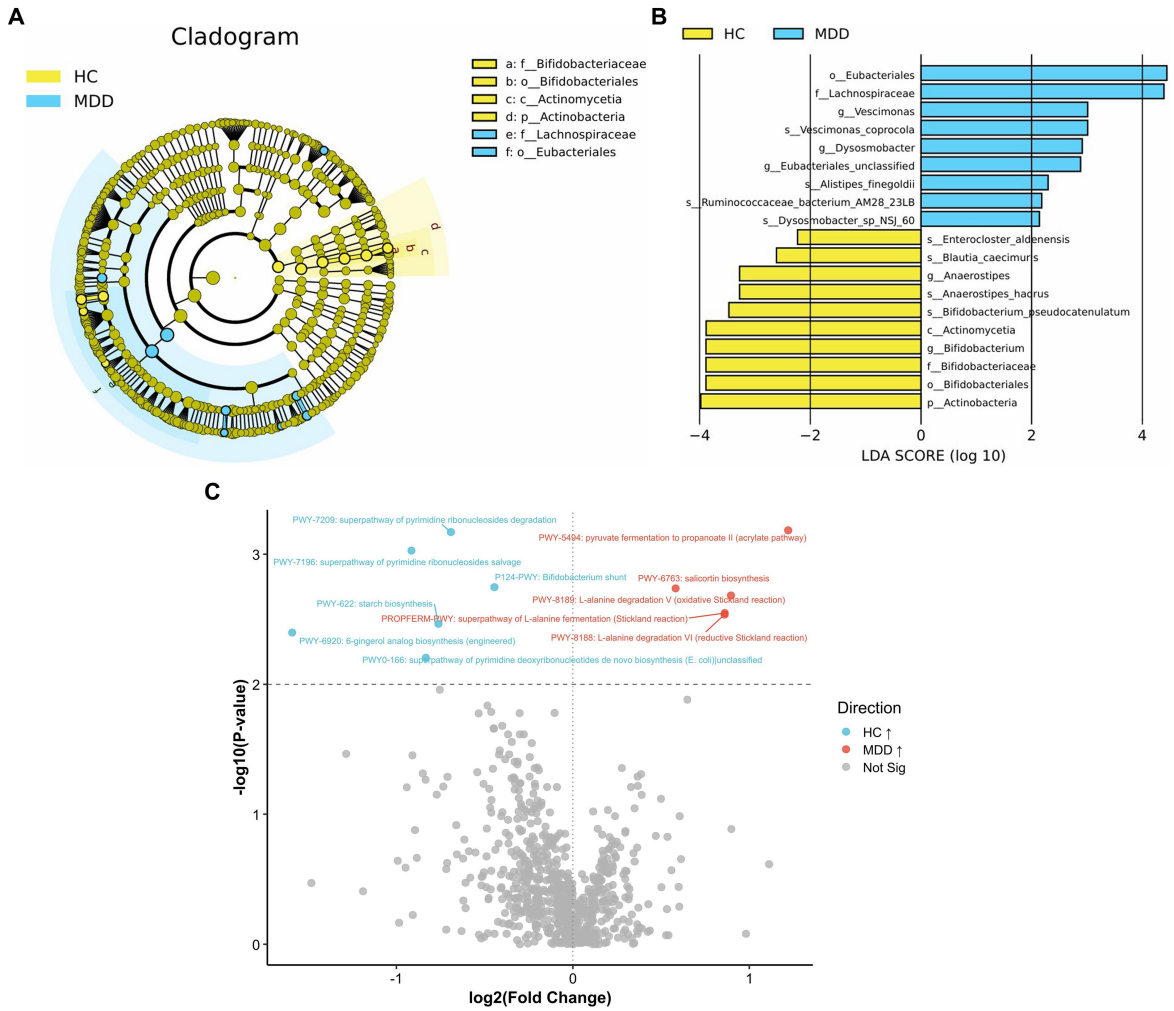
Differences in the composition of gut microbiome. **(A,B)** LEfSe analysis showing differences in the abundance of gut microbiome and **(C)** Volcano Plots indicating the variation of gut predicted metabolic pathways between MDD and HC group. p: Phylum, c: Class, o: Order, f: Family, g: Genus, s: Species.

The linear discriminant analysis (LDA) effect size (LEfSe) analysis is based on the LDA score for each species to reflect its contribution to intergroup differences. The species tree results indicated that *Lachnospiraceae* and *Eubacteriales* were more enriched in the MDD group, and they also contributed more significantly to the differences between the two groups (all *p* < 0.05; Figure 2A). In contrast, the relative abundance of *Actinobacteria* (phylum), particularly within the *Bifidobacteriaceae* family, was significantly higher in the gut microbiota of the HC group (all *p* < 0.05; Figure 2A). The bar chart displays other species with significant differences. The relative abundances of bacteria such as *Vescimonas*, *Vescimonas coprocola*, *Dysosmobacter*, *unclassified Eubacteriales*, *Alistipes finegoldii*, *Ruminococcaceae bacterium AM28-23LB*, and *Dysosmobacter* sp. *NSJ-60* in the MDD group were significantly higher than those in the HC group. Conversely, the relative abundances of bacteria such as *Enterocloster aldenensis*, *Blautia caecimurs*, *Anaerostipes*, *Anaerostipes hadrus*, *Bifidobacterium pseudocatenulatum*, *Actinomycetia*, and *Bifidobacterium* in the HC group were greater than those in the MDD group (Figure 2B).

Volcano plots also showed that some predicted metabolic pathways, such as pyruvate fermentation to propanoate II, salicortin biosynthesis, superpathway of L-alanine fermentation, and L-alanine degradation V and VI, were significantly enhanced in the MDD group in comparison with the HC group (Figure 2C). In contrast, the levels of six predicted metabolic pathways were upregulated in the HC group in comparison with the MDD group, including 6-gingerol analog biosynthesis, starch biosynthesis, *Bifidobacterium shunt*, and the superpathway of pyrimidine ribonucleoside degradation, pyrimidine ribonucleoside salvage, and pyrimidine deoxyribonucleotide *de novo* biosynthesis.

### 3.4 Correlation between the gut microbiota, the peripheral and cognitive function

**Figure 3:**
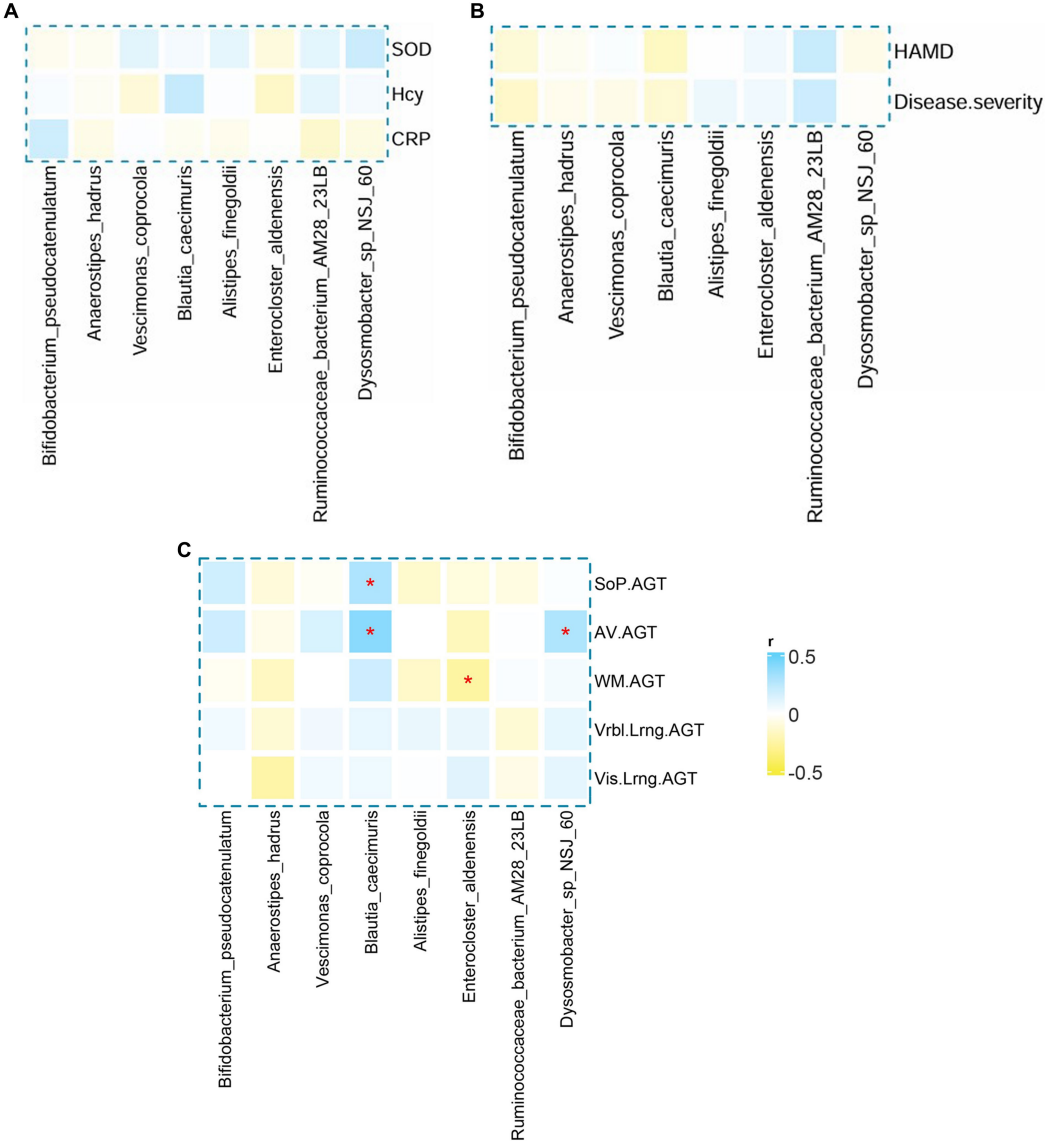
Correlation between gut microbiome and clinical traits. Correlation between gut microbiome and **(A)** peripheral blood indicators; **(B)** severity of disease and depressive symptoms; **(C)** cognitive levels with MCCB T scores. SOD: superoxide dismutase, CRP: C- reactive protein, HAMD: Hamilton Depression Scale score, SoP.AGT: processing speed, AV.AGT: attention/vigilance, WM.AGT: working memory, Vrbl.Lrng.AGT: verbal learning, Vis.Lrng.AGT: visual learning. Star symbols indicate that there is a significant correlation between the two indicators.

Subsequently, to investigate the associations between the differential gut microbiota, the related blood indicators and cognitive function, we conducted a correlation analysis in the MDD group on the basis of the differential microbiota between the MDD and HC groups. No significant correlation was observed between the relative abundance of the altered gut microbiota and clinical indicators (Figures 3A,B). However, the differential gut microbiota exhibited correlations with cognitive function based on the MCCB score in the MDD group. The relative abundance of *Blautia_caecimuris* showed significant positive correlations with processing speed (*r* = 0.295, *p* = 0.021) and the attention/vigilance domain (*r* = 0.408, *p* = 0.001). The relative abundance of *Dysosmobacter_sp._NSJ-60* showed a significant positive correlation with attention/vigilance domain (*r* = 0.290, *p* = 0.024). The relative abundance of *Enterocloster_aldenensis* and the T score for working memory (*r* = *−*0.257, *p* = 0.046) showed a statistically significant negative correlation (Figure 3C). All the specific values of the correlation analyses about gut microbiota are presented in the Supplementary Table S1. Besides, we conducted a correlation analysis between the related blood indicators and cognitive function in the MDD group. We found that the levels of CRP and the T score for working memory showed a statistically significant positive correlation (*r* = 0.373, *p* = 0.018) and the levels of SOD and the T score for verbal learning memory showed a statistically significant negative correlation (*r* = *−*0.327, *p* = 0.039). Other T scores of MCCB have no correlation with blood indicators (all *p* > 0.05).

### 3.5 Correlation between gut functional pathways and clinical traits

**Figure 4:**
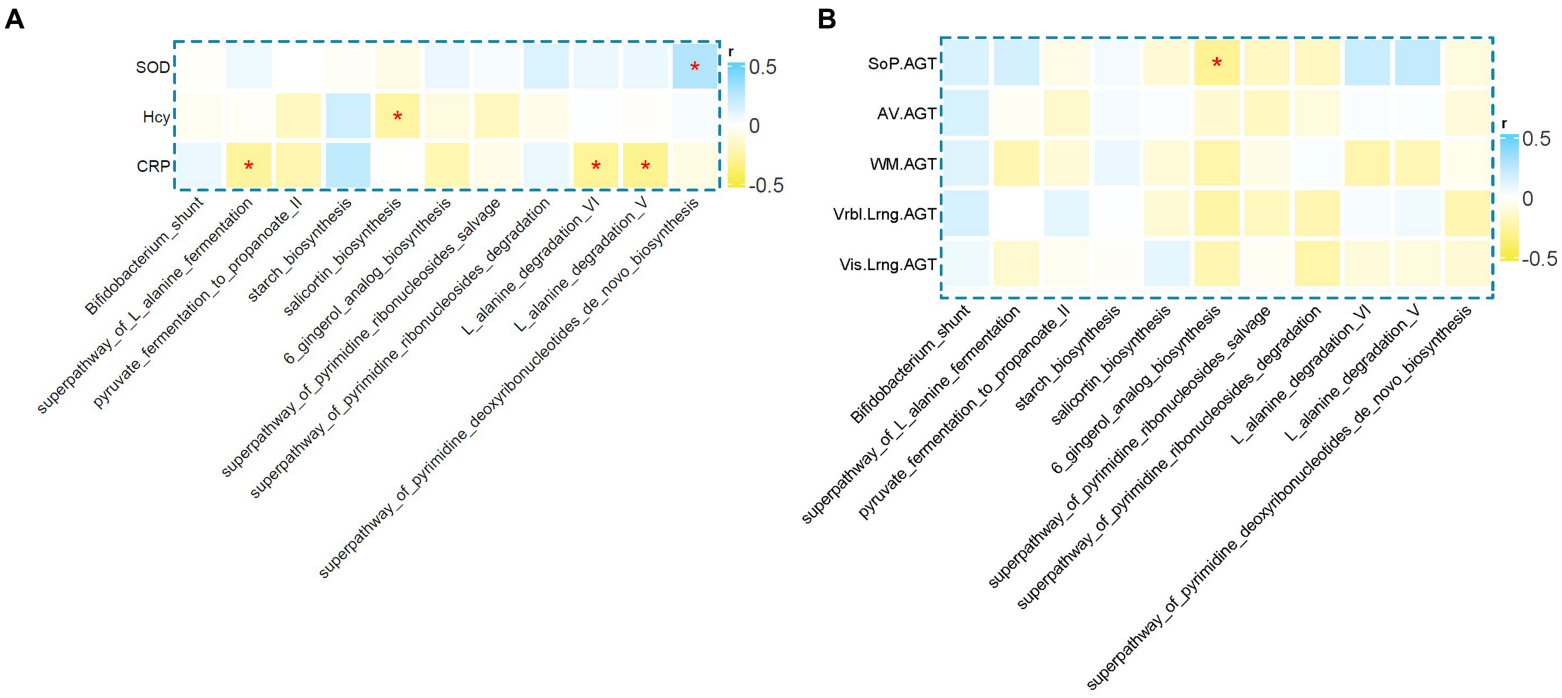
Correlation between differential pathways and clinical traits. Correlation between differential pathways and **(A)** peripheral blood indicators; **(B)** cognitive levels with MCCB T scores. SOD: superoxide dismutase, CRP: C- reactive protein, SoP.AGT: processing speed, AV.AGT: attention/vigilance, WM.AGT: working memory, Vrbl.Lrng.AGT: verbal learning, Vis.Lrng.AGT: visual learning. Star symbols indicate that there is a significant correlation between the two indicators.

Subsequently, we identified several significant correlations between the predicted pathway abundance of gut microbiota and clinical characteristics in the MDD group. A few functional pathways showed significant correlations with serum indicators. The relative abundance of salicortin biosynthesis was negatively correlated with the serum levels of Hcy (*r* = *−*0.255, *p* = 0.049; Figure 4A). The levels of pathways related to L-alanine metabolism, such as the superpathway of L-alanine fermentation and L-alanine degradation VI (*r* = *−*0.289, *p* = 0.025) and V (*r* = *−*0.306, *p* = 0.017), which were negatively correlated with the levels of CRP, showed differences between the MDD and HC groups (Figure 4A). The superpathway of pyrimidine deoxyribonucleotide *de novo* biosynthesis showed a significant positive correlation with the serum SOD activity (*r* = 0.279, *p* = 0.030; Figure 4A). Moreover, the 6-gingerol analog biosynthesis pathway showed a negative correlation with the T score for processing speed in the MCCB (*r* = *−*0.298, *p* = 0.020; Figure 4B). All the specific values of the correlation analyses are presented in the Supplementary Table S2.

## 4 Discussion

Our study aimed to investigate the relationships of cognitive impairment with peripheral OS and inflammatory markers as well as the gut microbiota and their associated predicted metabolic pathways in first-episode, drug-naïve patients with MDD. The results demonstrated that in comparison with HCs, patients with MDD exhibited substantial cognitive impairment, elevated SOD activity, enrichment of specific gut microbiota (e.g., *Lachnospiraceae* and *Eubacteriales*), and upregulated microbial predicted metabolic pathways involving alanine, pyruvate, and salicortin. The relative abundance of salicortin biosynthesis, the levels of pathways related to L-alanine metabolism, and the de novo biosynthesis of superpyrimidine deoxyribonucleotides were correlated with the levels of Hcy, CRP and SOD activity, respectively. Moreover, the 6-gingerol analog biosynthesis pathway was negatively correlated with the T score for processing speed. The T scores for some dimensions of the MCCB also showed correlations with the gut microbiota.

Our results showed that patients with MDD exhibited comprehensive impairment in cognitive function along with alterations in OS markers, gut microbiota, and predicted metabolic pathways, consistent with the results of previous studies (Huang et al., 2025; Zhang et al., 2025). However, the previous studies did not explore the relationships of these indicators and their possible roles. Moreover, some studies did not match patients and HCs by years of education. Furthermore, body weight differences among individuals can lead to variations in adipose tissue content, which may confound measures of OS, inflammation, and gut microbiota. In the present study, we further controlled for body weight to minimize the variability resulting from these factors. Our findings showed that the relative abundance of specific taxa—namely, the order *Eubacteriales*, genus *Vescimonas*, and species *Vescimonas coprocola* (which belong to the class Clostridia within the phylum *Bacillota*)—was higher in patients with MDD than in HCs. *Vescimonas* has been primarily implicated in the degradation of dietary fiber and complex carbohydrates, and it plays a key functional role in the production of butyrate—a major short-chain fatty acid (SCFA) (Salazar-Jaramillo et al., 2024). Butyrate has been extensively documented as a beneficial microbial metabolite, demonstrating critical support for host energy metabolism and a pronounced ability to enhance intestinal epithelial barrier integrity (Wieërs et al., 2019). Consequently, increased relative abundance of *Vescimonas* and *Vescimonas coprocola* may represent compensatory responses during the acute phase of first episode MDD. Meanwhile, we also observed an increase in the relative abundance of *Ruminococcaceae* and *Lachnospiraceae*, which demonstrated significant positive correlations with elevated levels of butyrate in the brain (Li et al., 2021). Since butyrate is known to exert anti-inflammatory effects (Nikolova et al., 2021), this finding indicates a compensatory increase in the levels of probiotics during the acute phase of the illness. Consistent with this interpretation, our hematological analysis revealed no marked elevation in the levels of the inflammatory marker CRP, which could be attributed to the anti-inflammatory regulatory action of butyrate produced by these compensatory microbial shifts. On the other hand, the elevated relative abundance of *Vescimonas* and the species *Vescimonas coprocola* could enhance carbohydrate utilization, potentially contributing to disturbances in carbohydrate metabolism linked to depressive symptomatology. Previous studies have associated dysregulation in carbohydrate and amino acid metabolism with the manifestation of depressive symptoms (Bastiaanssen et al., 2020; Zheng et al., 2016). Moreover, animal research has demonstrated that fecal microbiota transplantation from depressed patients into healthy rodents induces depression like behaviors, which are accompanied by disruptions in carbohydrate and amino acid metabolism (Kelly et al., 2016). Our findings also indicated an increased relative abundance of *Lachnospiraceae*, which actively impairs glucose metabolism and shows a positive correlation with the severity of depressive symptoms. Thus, the gut microbiota may influence depressive symptoms through its effects on carbohydrate metabolism. A recent study combining human research with animal experiments also supports our inference, showing that gut microbiota-driven host energy metabolism disorders mediate depressive behaviors, and that early autologous fecal microbiota transplantation can restore central energy balance, thereby alleviating depression (Lei et al., 2026). Consistent with the findings of previous studies (Wang et al., 2025), we also observed elevated relative abundances of *Alistipes* and *Dysosmobacter* in patients with MDD. *Alistipes* is an indole-positive organism capable of reducing serotonin bioavailability and converting glutamate to *γ*-aminobutyric acid (GABA) through glutamate decarboxylase expression; its increased relative abundance may disrupt gut–brain axis function (Parker et al., 2020). Although literature on *Dysosmobacter* is limited, previous studies have suggested it can modulate immune responses through interactions with the host immune system (Rong and Shu, 2024). In summary, patients with MDD exhibit disturbances in the gut microbiota, which may contribute to depressive symptoms through multiple pathways, including effects on carbohydrate metabolism, serotonin signaling, and inflammatory processes.

We also employed gut metagenomic sequencing to investigate the alterations in intestinal predicted metabolic pathways and their associations with peripheral blood biomarkers in patients with first-episode MDD. Our analysis revealed differences in microbial metabolic pathways between HCs and patients with MDD, including marked upregulation of pathways related to alanine metabolism, pyruvate fermentation to propanoate II, and salicortin biosynthesis. The first two pathways are closely linked to alanine metabolism and cellular energy supply. Recent studies have identified a significant association between higher carbohydrate intake and a reduced risk of MDD (Xie et al., 2024), further supporting our earlier hypothesis that gut microbiota dysbiosis contributes to depressive symptoms by disrupting carbohydrate and amino acid metabolism. Furthermore, the superpathway of L-alanine fermentation and L-alanine degradation V are explicitly associated with L-alanine metabolism, wherein the amino group of L-alanine is transferred to *α*-ketoglutarate to yield pyruvate and L-glutamate. Glutamate is widely distributed throughout the brain and serves as a major excitatory synaptic neurotransmitter (Mathews et al., 2012), playing key roles in regulating neuroplasticity, learning, and memory (Malenka and Nicoll, 1999). The results of targeted metabolomics combined with metagenomics also support our findings. They show that in patients with depression, the relative abundance of gut microbiota is associated with mitochondrial fatty acid synthesis, ketogenesis, and amino acid metabolism, as well as with altered levels of core metabolites such as L-glutamate (Lei et al., 2026). Glutamate levels in the plasma, serum, cerebrospinal fluid, and brain tissue have been linked to mood, psychiatric disorders, and suicidality (Frye et al., 2007). With a growing body of evidence implicating glutamate in the pathophysiology of MDD, it is rapidly emerging as a novel therapeutic target for this disorder. For instance, ketamine has been shown to enhance glutamate signaling in both rodents and humans (Chowdhury et al., 2017; Duman et al., 2019) and can induce a rapid reduction in depressive symptoms (McGirr et al., 2015). Additionally, studies have shown that alanine and glutamate are non-essential amino acids that exhibit a strong positive correlation with HAMD scores (Mitani et al., 2006). One study identified alanine as a significant discriminant factor for suicide attempts in patients with MDD (Zheng et al., 2013). Our results indicated a negative correlation between pathways related to alanine metabolism and CRP levels; however, the peripheral blood CRP levels in MDD patients did not show a significant increase or decrease in comparison with those in HCs. A previous cumulative meta-analysis reported that CRP levels are most closely associated with the severity of depressive symptoms and are highly correlated with symptoms of anhedonia and psychomotor retardation (Farooq et al., 2018; Haapakoski et al., 2015). Nevertheless, our findings did not reveal a significant correlation between depressive symptoms and CRP levels. These results support our hypothesis that patients with first episode MDD exhibit a compensatory increase in probiotic levels, which modulate the predicted metabolic pathways to counteract depressive pathophysiology. Meanwhile, alanine metabolism also plays an important role in maintaining redox balance. The increased expression of alanine related predicted metabolic pathways indicates a compensatory mechanism to alleviate inflammation induced by OS in patients with first episode MDD. Furthermore, our results showed a marked increase in the salicortin biosynthesis pathway in patients with MDD comparison with those in HCs. Salicortin, a bioactive phytochemical identified in the young twigs of S. pseudolasiogyne, is a salicylic acid derivative containing a 1 hydroxy 6 oxo 2 cyclohexenecarboxylic acid group that shows anti-inflammatory properties and inhibitory effects on adipogenesis (Kim et al., 2022; Kwon et al., 2014). In patients with MDD, enhanced salicortin biosynthesis may elevate salicortin production, thereby exerting an anti-inflammatory effect. This could partly explain why CRP and Hcy levels in patients with MDD did not increase significantly in comparison with those in HCs. Correlation analyses revealed a negative association between the salicortin biosynthesis pathway and Hcy levels. Hcy and its oxidative metabolites act as agonists of N-methyl-D-aspartate (NMDA) receptors *in vivo* (Moretti et al., 2021). Hcy stimulates NMDA receptors, increasing calcium influx and exerting neurotoxic effects (Ziemińska et al., 2003), and thereby altering neurotransmission and inducing neuronal excitotoxicity, apoptosis, *β* amyloid accumulation, and NMDA receptor over activation (Sharma et al., 2015). Finally, our study showed that peripheral blood SOD activity was significantly greater in patients with first episode MDD and had a positive correlation with the superpathway of pyrimidine deoxyribonucleotide *de novo* biosynthesis. In this pathway, the initial reactant glutamine is synthesized from glutamate by glutamine synthetase—a process accompanied by ammonia detoxification and redox reactions (Warner et al., 2014). SOD serves as a crucial endogenous antioxidant and plays an important role in detoxifying xenobiotics and their metabolites and in maintaining intracellular redox balance (Vairetti et al., 2021). Therefore, the interaction between SOD and the pyrimidine deoxyribonucleotide de novo biosynthesis superpathway may be linked through redox mechanisms. In summary, the gut microbiota in patients with first episode MDD may metabolically upregulate pathways related to OS and anti-inflammatory responses to counteract disease associated stress, while simultaneously potentially contributing to depressive symptoms by disrupting the balance of the excitatory neurotransmitter glutamate metabolism. However, since our study did not include blood metabolomic profiling, future studies should incorporate blood metabolomics to further validate these hypotheses.

This study used gut metagenomic analyses to explore the relationships of the gut microbiota and their predicted metabolic pathways with cognitive function in MDD. The novel finding in this study was the presence of a negative correlation between the 6-gingerol analog biosynthesis pathway and the processing speed cognitive domain. The primary product of this pathway is a 6-gingerol analog, and no studies to date have investigated the role of 6-gingerol analogs in MDD. Research on cognitive impairment in animal models has shown that samples with the highest 6-gingerol content can improve cognitive function by restoring neurotransmitter levels and modulating inflammatory and antioxidant parameters in mice with scopolamine-induced cognitive deficits (Ghosh et al., 2025). Besides, our results revealed that the SOD levels in patients with MDD were higher than those in healthy controls, indicating that the OS response is involved in the acute onset of depression, and it shows a compensatory increase. The research indicates that there is an activated inflammatory response system in MDD, and it shows a compensatory reaction during the acute phase. Mowever, other studies have indicated that 6-gingerol may impair mitochondrial function and lead to apoptosis. Treatment with 6-gingerol has been shown to induce considerable cytotoxicity, which is mediated by the generation of ROS (Nigam et al., 2009) that inhibit cell growth. Increased ROS levels cause a reduction in the mitochondrial membrane potential and subsequent induction of apoptosis, which is directly affecting synaptic plasticity and hippocampal neurons (Wang et al., 2024). Therefore, an increase in 6-gingerol analogs may promote neuronal apoptosis, thereby contributing to cognitive impairment in patients with MDD. The relative abundance of *Enterocloster aldenensis* showed a statistically significant negative correlation with the T score for working memory. *Enterocloster aldenensis*, which was initially described as *Clostridium aldenense*, is an anaerobic Gram-positive bacillus that plays a key role in maintaining gut health through the fermentation of complex carbohydrates (Magdy Wasfy et al., 2023). Despite its commensal nature, *E. aldenensis* has been identified in clinical cases, including cases of bacteremia and intra-abdominal infections. It may contribute to cognitive impairment by triggering inflammatory responses through occult infection. Besides, our correlation results show that the levels of CRP is positively correlated with the T score for working memory, which also supports this inference. This might be related to the compensatory activation under emergency conditions. During the acute phase of MDD, the activated inflammatory response system will simultaneously trigger the activation of the compensatory immune response system, in order to exert anti-inflammatory effects and maintain immune balance (Debnath et al., 2021). Conversely, the relative abundance of *Dysosmobacter* sp. *NSJ-60* and *Blautia caecimuris* showed significant positive correlations with the attention/vigilance domain and the processing speed dimension and attention/vigilance domain, respectively. *Blautia* is considered to perform a protective role in a wide range of disease states, and its relative abundance is reduced in patients with colorectal cancer, inflammatory bowel disease, cirrhosis, and obesity (Liu X. et al., 2021). *Blautia* species, which are typically associated with a healthy state, are major producers of SCFAs (Koh et al., 2016). As a genus most closely linked to the regulation of intestinal inflammation, atherosclerosis, and immune system maturation, *Blautia* mediates its effects through its metabolic end product, butyrate (Kasahara et al., 2018). *Dysosmobacter* sp. *NSJ-60* is another bacterium known for butyrate production. SCFAs, particularly butyrate, serve as a primary energy source for colonic epithelial cells (Goverse et al., 2017) and play key roles in modulating the surrounding microbial environment and directly interacting with the host immune system (Steinmeyer et al., 2015). Butyrate can downregulate the production of pro-inflammatory cytokines such as interleukin (IL)-1β, IL-6, and IL-8 (Cleophas et al., 2016). Furthermore, butyrate is associated with enhanced mitochondrial activity (Cani and Van Hul, 2024). Since inflammation and OS are known to impair cognitive function, future studies should consider exploring the effectiveness of increasing the relative abundance of such probiotics to improve cognitive function in patients with MDD.

However, this study still had the following limitations: (1) this was a cross-sectional study. Thus, the relationships between the gut microbiota and their predicted metabolic pathways and cognitive function as well as peripheral blood indicators can only be considered to indicate correlation, not causation. (2) Assessments of intestinal predicted metabolic pathways can only indicate differences in these pathways. The specific relationship between the action sites and blood metabolites remains unclear. Thus, future studies should incorporate blood metabolomics analyses for verification. (3) Due to the relatively small sample size and the lack of sufficient data to identify microbiome or metabolite features that are simultaneously correlated with clinical indicators, there is insufficient basis for conducting mediation or network analyses. Further complex analyses would require larger sample sizes and more clinical data to support them.

Patients with first episode MDD showed alterations in cognitive function, SOD activity, and the gut microbiota and their predicted metabolic pathways. The gut microbiota and their predicted metabolic pathways showed close correlations with cognitive performance and peripheral blood indicators, including SOD activity and Hcy and CRP levels. In these patients, the gut microbiome may metabolically upregulate pathways related to OS and anti-inflammatory responses to counteract disease-associated stress while potentially contributing to depressive symptoms by disrupting the balance of excitatory neurotransmitter glutamate metabolism. The probiotics *Dysosmobacter* sp. *NSJ-60* and *Blautia* were significantly associated with cognitive function. Thus, future interventions targeting cognitive impairment in MDD may involve probiotic supplementation rich in *Dysosmobacter* sp. *NSJ-60* and *Blautia*. However, since our study was cross-sectional and did not include blood metabolomics data, these conclusions can only establish correlations rather than causation. Therefore, future studies should incorporate blood metabolomics assessments to further validate these hypotheses.

## Data Availability

The datasets generated and analyzed during the current study are not publicly available due to institutional and ethical restrictions related to data security and participant confidentiality. Datasets are available from the corresponding author upon reasonable request.
